# What is mutation? A chapter in the series: How microbes “jeopardize” the modern synthesis

**DOI:** 10.1371/journal.pgen.1007995

**Published:** 2019-04-01

**Authors:** Devon M. Fitzgerald, Susan M. Rosenberg

**Affiliations:** 1 Department of Molecular and Human Genetics, Baylor College of Medicine, Houston, Texas, United States of America; 2 Department of Biochemistry and Molecular Biology, Baylor College of Medicine, Houston, Texas, United States of America; 3 Department of Molecular Virology and Microbiology, Baylor College of Medicine, Houston, Texas, United States of America; 4 The Dan L Duncan Comprehensive Cancer Center, Baylor College of Medicine, Houston, Texas, United States of America; Dalhousie University, CANADA

## Abstract

Mutations drive evolution and were assumed to occur by chance: constantly, gradually, roughly uniformly in genomes, and without regard to environmental inputs, but this view is being revised by discoveries of molecular mechanisms of mutation in bacteria, now translated across the tree of life. These mechanisms reveal a picture of highly regulated mutagenesis, up-regulated temporally by stress responses and activated when cells/organisms are maladapted to their environments—when stressed—potentially accelerating adaptation. Mutation is also nonrandom in genomic space, with multiple simultaneous mutations falling in local clusters, which may allow concerted evolution—the multiple changes needed to adapt protein functions and protein machines encoded by linked genes. Molecular mechanisms of stress-inducible mutation change ideas about evolution and suggest different ways to model and address cancer development, infectious disease, and evolution generally.

## Introduction

Mutation is any change in the sequence of an organism’s genome or the process by which the changes occur. Mutations range from single-basepair alterations to megabasepair deletions, insertions, duplications, and inversions. Though seemingly simple, ideas about mutation became entangled with the initially simplifying assumptions of both Darwin himself and the “Modern Synthesis”—the geneticists who embraced Darwin in the pre-DNA early 20th century, beginning evolutionary biology. The assumptions of purely “chance” mutations that occur constantly, gradually, and uniformly in genomes have underpinned biology for almost a century but began as a “wait-and-see”–based acknowledgment by early evolutionary biologists that they did not know the chemical nature of genes or how mutations in genes might occur.

Darwin considered generation of variation by chance to be a simplifying assumption, given that the origins of variation (and genes!) were unknown in his time, but he appears to have thought chance variation to be unlikely: “I have hitherto sometimes spoken as if the variations—so common and multiform in organic beings under domestication, and in a lesser degree in those in a state of nature—had been due to chance. This, of course, is a wholly incorrect expression, but it serves to acknowledge plainly our ignorance of the cause of particular variation [Chapter 5, 1].”

He also described multiple instances in which the degree and types of observable variation change in response to environmental exposures, thus seeming open to the possibility that the generation of variation might be environmentally responsive [1]. However, even once mutations were described on a molecular level, many continued to treat spontaneous mutations as necessarily chance occurrences—typically as mistakes occurring during DNA replication or repair. Darwinian evolution, however, requires only two things: heritable variation (usually genetic changes) and selection imposed by the environment. Any of many possible modes of mutation—purely “chance” or highly biased, regulated mechanisms—are compatible with evolution by variation and selection.

Here, we review some of the wealth of evidence, much of which originated in microbes, that reframes mutagenesis as dynamic and highly regulated processes. Mutation is regulated temporally by stress responses, occurring when organisms are poorly adapted to their environments, and occurs nonrandomly in genomes. Both biases may accelerate adaptation.

## Bacteria teach biologists about evolution

Microbes were initially held as proof of the independence of mutational processes and selective environments. The Luria–Delbruck experiment (1943) demonstrated that bacterial mutations to phage resistance can occur prior to phage exposure [2], and the Lederbergs showed similar results for resistance to many antibiotics [3]. However, discovery of the SOS DNA-damage response and its accompanying mutagenesis [4–7] in the post-DNA world of molecular genetics began to erode the random-mutation zeitgeist. Harrison Echols thought that the SOS response conferred “inducible evolution” [8], echoing Barbara McClintock’s similar SOS-inspired suggestion of adaptation by regulated bursts of genome instability [9]. But SOS mutagenesis might be an unavoidable byproduct of DNA repair, and high-fidelity repair might be difficult to evolve, many argued. John Cairns’ later proposal of “directed” or “adaptive” mutagenesis in starvation-stressed *Escherichia coli* [10, 11] reframed the supposed randomness of mutation as an exciting problem not yet solved. The mutagenesis they studied under the nonlethal environment of starvation is now known to reflect stress-induced mutagenesis—mutation up-regulated by stress responses. Its molecular mechanism(s), reviewed here, demonstrate regulation of mutagenesis. Similar mechanisms are now described from bacteria to humans, suggesting that regulated mutagenesis may be the rule, not the exception (discussed here and reviewed more extensively, [12]).

### Stress-induced mutagenic DNA break repair in *E*. *coli*

DNA double-strand breaks (DSBs) occur spontaneously in approximately 1% of proliferating *E*. *coli* [13, 14]. In unstressed *E*. *coli*, DSB repair by homologous recombination (HR) is relatively high fidelity. However, activation of the general stress response, for example, by starvation, flips a switch, causing DSB repair to become mutagenic [15, 16]. This process of mutagenic break repair (MBR) causes mutations preferentially when cells are poorly adapted to their environment—when stressed—and, as modeling indicates [17–20], may accelerate adaptation.

At least three stress responses cooperate to increase mutagenesis in starving *E*. *coli*. The membrane stress response contributes to DSB formation at some loci [21]; the SOS response up-regulates error-prone DNA polymerases used in one of two MBR mechanisms [22–24]; and the general stress response licenses the use of, or persistence of errors made by, those DNA polymerases in DSB repair [15, 16]. The requirement for multiple stress responses indicates that cells check a few environmental conditions before flipping the switch to mutation [25]. *E*. *coli* MBR is a model of general principles in mutation from bacteria to human: the regulation of mutation in time, by stress responses, and its restriction in genomic space, limited to small genomic regions, in the case of MBR, near DNA breaks. We look at MBR, then other mutation mechanisms in microbes and multicellular organisms, which share these common features.

### MBR mechanisms

**Fig 1:**
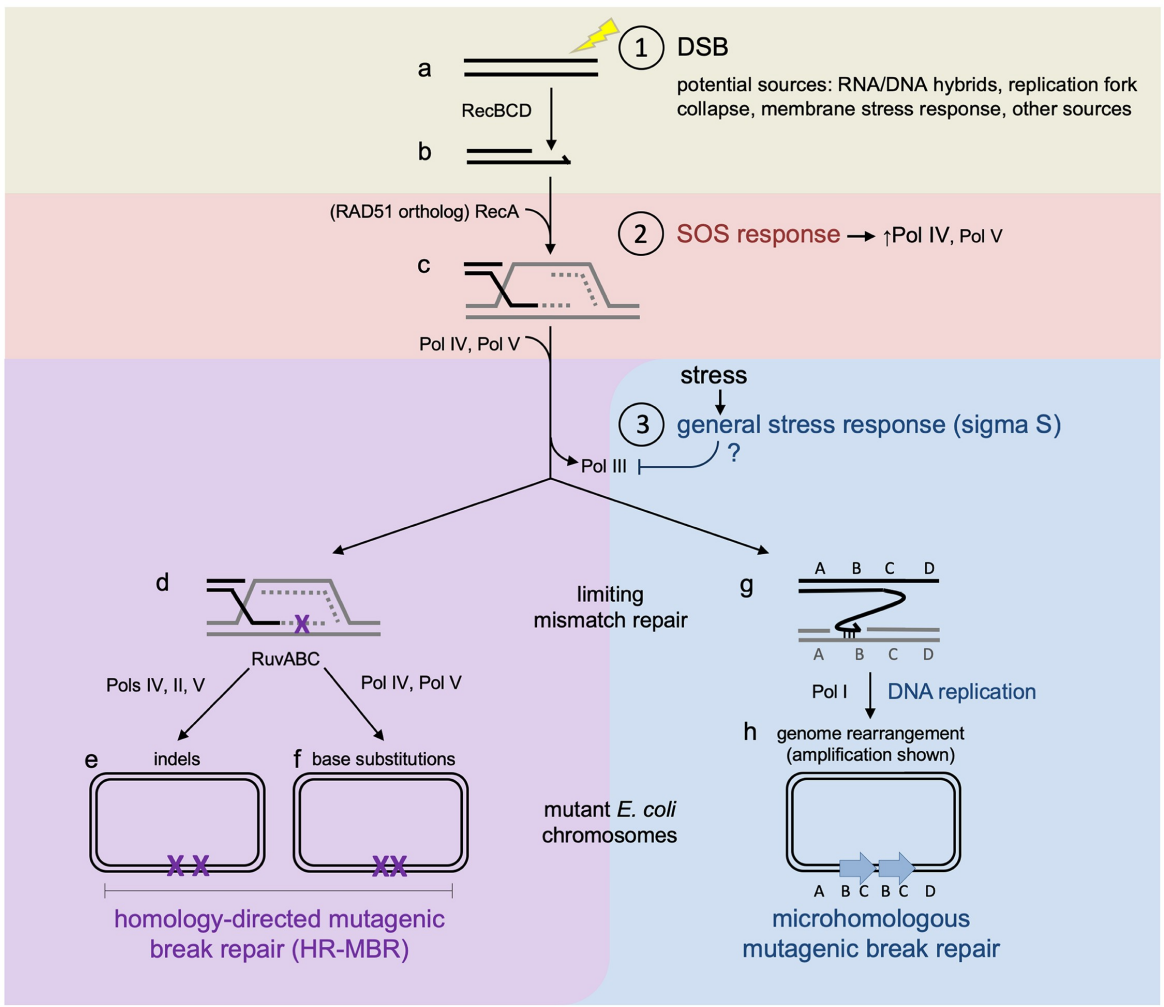
*E*. *coli* MBR models. (a–c) RecBCD nuclease loads RecA HR protein onto ssDNA, similarly to human BRCA2 loading RAD51; basepairing with a strand of identical duplex DNA (gray, e.g., a sister chromosome). Parallel lines, basepaired DNA strands. Repair synthesis (dashed lines) is switched to a mutagenic mode by the general stress response (sigma S). DNA polymerase errors (d, purple X) generate indels (e, purple XX) and base substitutions (f, purple XX). Microhomologous MBR requires DNA Pol I for template switching to regions containing microhomology (g), of as little as a few basepairs, and initiates replication, creating genome rearrangements; (h) a duplicated chromosome segment (blue arrows) is shown here. Circled numbers and shading indicate the three main events in HR-MBR: ① a DSB and its repair by HR, ② the SOS response (pink), and ③ the general stress response (blue). Note that HR-MBR (d–f, purple) requires both the SOS response (②, pink, which up-regulates error-prone DNA Pol IV, necessary for HR-MBR) and general stress response (③, blue), but microhomologous MBR (g–h, blue) requires the general stress response but not SOS (③, blue). Figure modified from [12]. HR, homologous recombination; MBR, mutagenic break repair; ssDNA, single-stranded DNA.

Two distinct but related MBR mechanisms occur in starving *E*. *coli*, and both require activation of the general/starvation response. Moreover, both occur without the starvation stress if the general stress response is artificially up-regulated [15, 16], indicating that the stress response itself without actual stress is sufficient. Homologous-recombinational (homology-directed) MBR (HR-MBR) generates base substitutions and small indels via DNA-polymerase errors during DSB-repair synthesis (Fig 1A–1F). Microhomologous MBR causes amplifications and other gross chromosomal rearrangements (GCRs) [26–28], most probably by microhomology-mediated break-induced replication (MMBIR) [28, 29] (Fig 1A–1C, 1G and 1H). Both MBR pathways challenge traditional assumptions about the "chance" nature of mutations.

Both MBR mechanisms are initiated by a DSB and require HR DSB-repair proteins (Fig 1, ①) [15, 28, 30–33]. The first steps mirror standard HR DSB repair: RecBCD nuclease processes DSB ends and loads RecA HR protein (Fig 1A and 1B). Next, the RecA–DNA nucleoprotein filament can activate the SOS response (Fig 1, ② pink), which is required for HR-MBR but not microhomologous MBR. RecA also facilitates strand invasion—the initial contact between the broken DNA molecule and an identical sister chromosome from which repair is templated (Fig 1C). In unstressed cells, this intermediate leads to high-fidelity HR repair; however, if the general stress response is activated, repair proceeds via one of two mutagenic pathways (Fig 1D–1H, ③). In HR-DSB repair, errors generated by error-prone SOS-up-regulated DNA polymerases IV (DinB), V (UmuDC), and II (PolB) accumulate in the tracts of repair synthesis during HR repair (Fig 1D) [22, 23, 34]. Activation of the general stress response licenses the use of these polymerases and/or prevents the removal of errors they generate: base substitutions and small indels (Fig 1E, 1F) [35, 36] that are located mostly in clusters/hotspots of about 100 kb around the original DSB location [30]. Microhomologous MBR requires DNA Pol I, which is proposed to promote microhomology-dependent template switching during repair synthesis to generate GCRs (Fig 1G and 1H) [28]. Similar MMBIR mechanisms are proposed to underlie many DSB-driven GCRs in human genetic diseases and cancers [28, 29, 37].

### Stress response regulation of *E*. *coli* MBR

Environmentally responsive and temporally regulated MBR mechanisms challenge long-held assumptions about the constant, gradual nature of mutagenesis and its blindness to an organism’s environmental suitability, or the lack of it, showing that mutagenesis is regulated tightly via environmental inputs. The general stress response controls the switch between high-fidelity or mutagenic DSB repair [15, 16]. This stress response, controlled by the alternative sigma factor σ^S^, is activated by starvation, cold, acid, antibiotic, oxidative, and osmotic stresses, among others. During a general stress response, the σ^S^ transcriptional activator increases the transcription of hundreds of genes (approximately 10% of all *E*. *coli* genes) that provide a range of protective functions (reviewed, [38]). We do not know exactly how the general stress response promotes mutagenesis. Two possibilities are as follows. First, the general stress response modestly up-regulates error-prone Pol IV above SOS-induced levels [39]. This might be the rate-limiting step. Also, the general stress response down-regulates mismatch repair (MMR) enzymes MutS and MutH [40, 41]. The HR-MBR mutation spectrum is similar to that of unstressed MMR-deficient strains [35, 36, 42], suggesting that MMR becomes limiting transiently during HR-MBR [36, 43, 44]. Other σ^S^ targets are also plausible, including down-regulation of the high-fidelity replicative DNA Pol III. Together, these observations suggest a model in which the general stress response enables error-prone polymerases to participate in DSB repair and/or allows the errors introduced by these polymerases to escape mismatch repair.

At least two other stress responses also contribute to one or both MBR mechanisms. The SOS DNA-damage response is required for HR-MBR [45] but not microhomologous MBR [22]. The SOS response is detected in about 25% of cells with a reparable DSB [13] and so comes automatically with the DSB that initiates MBR. (The 75% without SOS may repair fast enough to avoid SOS [13].) The SOS response halts cell division and activates DNA-damage tolerance and repair pathways. The primary role of the SOS response in HR-MBR is the upregulation of the error-prone DNA polymerases IV and V and possibly II. In some assays, production of Pol IV completely restores mutagenesis in SOS-defective cells [23]. In others, Pols II and V also contribute to mutagenesis [16, 34, 46]. Finally, the membrane stress response, regulated by σ^E^, promotes MBR at some loci by playing a role in spontaneous DSB formation through an unknown mechanism (see “Localization of MBR-dependent mutations”) [21]. The membrane stress response is triggered by an accumulation of unfolded envelope proteins caused by heat and other stressors [47] and therefore appears to couple these stressors to mutagenesis.

A genome-wide screen revealed a network of 93 genes required for starvation stress–induced MBR [25]. Strikingly, over half participate in sensing or signaling various types of stress and act upstream of activation of the key stress response regulators, which are hubs in the MBR network. During starvation stress, at least 31 genes function upstream of (in activation of) the general stress response. Most encode proteins used in electron transfer and other metabolic pathways, suggesting that these may be the primary sensors of starvation stress. Additionally, at least six genes are required for activation of the SOS response during MBR, and at least 33 MBR-network genes are required for activation of the membrane stress response. The 93 MBR genes form a highly connected network based on protein–protein interactions with the three stress response regulators (σ^S^, RecA/LexA, and σ^E^) as nonredundant network hubs [25]. The MBR network highlights the importance of tight, combinatorial stress response regulation of mutagenesis in response to multiple inputs.

### Generality of general stress response–promoted mutation

In *E*. *coli*, σ^S^-dependent mutagenesis has a mutational signature that is distinct from that seen in low-stress mutation accumulation (MA) studies and generation-dependent mutagenesis [34, 35, 42, 48]. Importantly, the nucleotide diversity in genomes of extant *E*. *coli* and other bacteria is described better by the σ^S^-dependent signature than the signature seen in MA studies [48]. Specifically, both σ^S^-dependent mutations and those seen in extant species have much higher ratios of transitions to transversions than is seen in MA experiments or expected by chance. This suggests that a significant portion of adaptive mutations in bacteria arise from σ^S^-dependent stress-induced mutation mechanisms such as MBR [48]. Furthermore, mathematical modeling suggests that stress response–regulated mutagenesis, such as MBR, promotes adaptation in changing environments [17–20]. Organisms that encode regulated mutagenesis mechanisms may have an increased ability to evolve, which would promote the evolution and maintenance of such mechanisms by second-order selection [17, 19, 20].

### Localization of MBR-dependent mutations

MBR generates mutations in hotspots close to the site of the instigating DSB, not at random locations in the genome [30, 49]. Hotspotting near DSBs is best described for HR-MBR initiated by engineered DSBs at various sites in the bacterial chromosome [30]. Mutations are most frequent within the first kilobase (kb) pair on either side of the DSB, and then fall off to near background levels approximately 60 kb from the break, with a weak long-distance hot zone of around 1 MB from the DSB site. This pattern of mutations supports the model that most MBR-dependent mutations arise from DNA polymerase errors during HR repair synthesis, and the remainder arise during more processive error-prone break-induced replication. The observation that mutations occur near DSBs does not, in itself, suggest that mutations are more likely to occur in certain genomic regions or in locations related to an organism’s adaptive “need.” However, it does suggest that the distribution of mutations is likely to mirror the distribution of DSBs, and the following lines of evidence suggest that DSB distributions may be nonrandom and reflect potential utility of genes in particular environments.

The sources and distributions of spontaneous DSBs are poorly understood in all organisms (reviewed, [14]), but we have some clues about the origins of DSBs that lead to MBR. First, transcriptional RNA–DNA hybrids (R-loops) are one source of MBR-promoting DSBs [50]. R-loops have been implicated in DSB formation in many experimental systems, although the exact mechanism(s) of DNA breakage is unresolved (reviewed, [51]). Though the distribution of R-loops has not been thoroughly assessed in starving *E*. *coli*, R-loops tend to be biased toward highly transcribed genes, promoters, and noncoding-RNA genes [52–54] and might, therefore, target DSBs and mutations to those sites. Also, activation of the σ^E^ membrane stress response is required for DSB formation in some assays and might target DSBs in genomic space [21]. The mechanism by which the σ^E^ stress response causes DSBs is unknown, but one possibility is that σ^E^-activated transcription causes DSBs directly (rather than via gene products’ up- or down-regulation), via an R-loop–dependent or other transcription-dependent mechanism. R-loops and the σ^E^ stress response might direct DSBs, and thus mutations, to regions of the genome with more adaptive potential for a given environment: transcribed genes and regulatory elements (promoters and regulatory small RNAs).

Additionally, MBR-dependent mutations can occur in clusters [55]. When a MBR-induced mutation occurs, the probability of finding another mutation at neighboring sites 10 kb away is approximately 10^3^ times higher than if the first mutation did not occur [55], and this is not true for a distant unlinked site in the genome [43], indicating that nearby mutations are not independent events. That is, linked mutations appear to occur simultaneously, in single MBR events. Such clusters are predicted to promote concerted evolution by simultaneously introducing changes to multiple domains of a protein or subunits of a complex protein machine [15, 20, 55]. Because multiple mutations are often needed for new functions to emerge, and often, the intermediate mutated states are less fit and counter selected, how complex protein machines evolve has been a long-standing problem [56]. Similar clusters have been identified in many organisms [57] and in cancer genomes, in which mutation clusters are called *kataegis*, Greek for (mutation) storms [58–60]. The mechanisms of mutation localization and co-occurrence revealed by MBR in *E*. *coli* have guided more mechanistic understanding of how mutation clusters occur across the tree of life.

Analyses of *E*. *coli* mutation accumulation lines and natural isolates indicate that local mutation rates vary by about one order of magnitude on the scale of approximately 10–100 kb [61, 62]. It is possible, even likely, that the DSB-dependent mutation localization and co-occurring mutation clusters characteristic of MBR are important contributors to this nonuniformity in mutation rate. Similar degrees of variation in local mutation rates have been reported for other bacteria [63], yeast [64], and mammals (mouse, human, and other primates [65, 66]) and could also result from MBR-like mutation mechanisms. Further analysis of natural isolates, with a specific focus on identifying clusters of cosegregating single-nucleotide variants, could indicate how frequent MBR-dependent mutation clusters are and how they shape genomes.

The molecular mechanisms of MBR reveal many ways by which mutations do not occur uniformly or independently from one another in genomic space. More work is needed to assess fully whether the MBR mechanism or genomes themselves have evolved to bias mutations to locations where they are most likely to be beneficial, such as genes actively transcribed in response to the experienced stressor.

## Other regulated mutagenesis mechanisms in microbes

In addition to starvation-induced MBR in *E*. *coli*, diverse bacteria and single-celled eukaryotes display examples of stress response–up-regulated mutagenesis. Some of these mutation mechanisms provide additional insight into how mutation rates vary across genomes in ways that may accelerate adaptive evolution. Many share characteristics with *E*. *coli* MBR but differ enough to suggest that regulated mutagenesis has evolved independently multiple times, thus highlighting the importance of regulated mutagenesis to evolution-driven problems, such as combatting infectious disease and antimicrobial resistance. Potential strategies to counteract pathogen evolution require understanding of how genetic variation is generated in these organisms. Continued study of regulated-mutagenesis mechanisms may reveal potential new drug targets to block mutagenesis and thus evolution [12, 25, 67].

### Other mechanisms of starvation stress–induced mutagenesis in bacteria

Diverse wild *E*. *coli* isolates show increased mutation rates during extended incubation on solid medium compared with vegetative growth, known as mutagenesis in aging colonies (MAC) [68]. In the one isolate tested for genetic requirements, MAC required σ^S^, decreased MMR capacity and error-prone Pol II but not DSB-repair proteins or SOS activation [68]—like, but not identical to, MBR in *E*. *coli*. *Bacillus subtilis* undergoes starvation-induced mutagenesis that is up-regulated by the ComK starvation-stress response and requires the SOS-induced Pol IV homolog YqjH but does not require DSB repair [69, 70]. In *B*. *subtilis*, starvation-induced mutation of reporter genes increases with increased levels of transcription of those genes, dependently on the transcription-coupled repair factor Mfd [71], similarly to *E*. *coli* MBR [50]. This suggests that transcription directs starvation-induced mutations to transcribed regions of the *B*. *subtilis* genome, where they are more likely to be adaptive. This is similar to the hypothesized targeting of *E*. *coli* MBR but occurs through a DSB-independent mechanism.

### Antibiotic-induced mutagenesis in bacteria

Many antibiotics, especially at subinhibitory concentrations, increase mutation rate and generate de novo resistance and cross-resistance in a variety of bacteria, including important pathogens. The β-lactam antibiotic ampicillin induces mutagenesis in *E*. *coli*, *Pseudomonas aeruginosa*, and *Vibrio cholera* via a mechanism requiring σ^S^, Pol IV, and limiting mismatch repair [41]. Whether DSBs are involved remains untested. The topoisomerase-inhibiting antibiotic ciprofloxacin (cipro) induces cipro resistance rapidly in *E*. *coli*, requiring HR proteins, SOS induction, and error-prone Pols II, IV, and V [72]. A requirement for σ^S^ has only very recently been demonstrated, along with the demonstration that cipro-induced mutagenesis is σ^S^-dependent MBR, similar to that induced by starvation[73]. In fact, diverse antibiotics both create DSBs [74] and activate the general stress response in *E*. *coli* [41], suggesting that these antibiotics may increase mutagenesis both by increasing DNA damage and triggering a switch to low-fidelity repair of that damage.

### Stress response regulation of mobile DNA elements in bacteria

Environmental stress up-regulates the activity of mobile DNA elements in many organisms, and this inducible genome instability is likely to be an important driver of evolution (reviewed, [75]). Although the mechanisms of regulation are poorly understood, stress response regulators have been implicated in a few cases. The general stress response promotes excision of an *E*. *coli* transposable prophage [76] and a *Pseudomonas* transposon [77]. Starvation increases the retromobility of *Lactobacillus lactis* LtrB group II intron through signaling by the small molecule regulators guanine pentaphosphate (ppGpp) and cyclic adenosine monophosphate (cAMP) [78]. Mobility of an *E*. *coli* transposon is increased by metabolic disruptions and negatively regulated by the σ^E^ membrane stress response [79]. Also, stress can directly regulate mobile element activity without an intervening stress response: movement of the T4 *td* intron becomes promiscuous during oxidative stress through ROS-induced oxidation of an amino acid in the intron-encoded homing endonuclease, which makes it a transposase [80].

### Regulated mutagenesis in eukaryotic microbes

Many examples of stress-associated mutagenesis and MBR have been reported in yeast, but stress response regulation has been demonstrated in only two cases. First, in the budding yeast *Saccharomyces cerevisiae*, the proteotoxic drug canavanine induces mutagenesis dependently on the MSN environmental stress response [81]. MSN-dependent mutagenesis requires the nonhomologous end-joining (NHEJ) protein Ku and two error-prone polymerases, Rev1 and Pol zeta (ζ) [81]. NHEJ is a relatively genome-destabilizing DSB-repair pathway, so MSN-dependent mutagenesis represents a stress-induced switch to MBR. NHEJ proteins are required for starvation-induced mutations in yeast as well [82]. Others have reported yeast MBR dependent on the error-prone DNA polymerase Rev3 [83] and spontaneous mutations dependent on error-prone polymerases Rev1 and Pol ζ [84]. Yeast also form mutation clusters by MBR [85] and undergo MMBIR similar to *E*. *coli* microhomologous MBR [86]. It is unknown whether these observations represent one or more mechanisms of mutation and whether MSN or other stress responses regulate mutagenesis in these cases. In all cases of yeast MBR, mutations are likely to occur near DSBs and, therefore, may be localized within genomes, as discussed for *E*. *coli* MBR.

Second, a heat shock response, activated by heat shock or protein denaturation, induces aneuploidy in *S*. *cerevisiae* by titration of the chaperone heat shock protein 90 (HSP90) [87]. Inhibitors of HSP90, such as radicicol, also induce aneuploidy. HSP90 is required for proper folding of kinetochore proteins in unstressed cells, so HSP90 titration or inhibition probably triggers aneuploidy through the disruption of kinetochore assembly [87]. The resulting yeast cell populations show high karyotypic and phenotypic variation and harbor cells resistant to radicicol and other drugs [87]. Aneuploidy in the form of extra chromosome copies may also facilitate adaptive evolution by providing a larger mutational target. Extra chromosomes may also buffer otherwise deleterious mutations through the sharing of gene products. Similar heat- and other stress-induced aneuploidy has been reported in *Candida albicans* and other yeast species, and can cause resistance to a variety of compounds, including clinically relevant antifungal drugs (reviewed, [88]). Some of these examples are likely to result from HSP90 titration, but other stress responses may be involved also.

## Regulated mutagenesis in multicellular organisms

Although microbes led the way in revealing mechanisms of stress response–up-regulated mutagenesis, many microbial mutation mechanisms are mirrored throughout the tree of life, including in multicellular organisms. Stress response–up-regulated mutation mechanisms have been discovered in plants, flies, and human cells (reviewed, [12]). The potential adaptive roles of these mutation mechanisms are less clear in multicellular organisms than in microbes. Do these mechanisms contribute to germline variation (and thus organismal evolution), mosaicism and somatic cell evolution, or both? Or are they simply biproducts of other required cellular functions or stress-induced dysfunctions?

In the *Drosophila* germline, the HSP90 heat shock response increases transposon-mediated mutagenesis and can drive organismal adaptation [89]. Most other regulated mutation mechanisms characterized to date have been in somatic cells, in which they might contribute to mosaicism. Somatic diversity may be important during development and contribute to organismal fitness, as is the case with antibody diversification during B-cell maturation. For example, neural development might require genetic complexity and plasticity as organisms get differently “wired” during development, based on their experiences. However, up-regulated mutagenesis is also likely to drive pathogenic somatic evolution, such as during cancer development. For example, hypoxic stress responses trigger down-regulation of mismatch repair and down-regulate HR DSB-repair proteins RAD51 and BRCA1, leaving only chromosome-rearranging nonhomologous or microhomologous DSB-repair mechanisms (reviewed, [90]). Hypoxic stress response–induced mutagenesis occurs in mouse and human, suggesting an adaptive function in addition to its probable relevance to tumor biology. Tumors become hypoxic and induce hypoxic stress responses, which promote angiogenesis. Hypoxic stress responses may also promote tumor evolution via mutagenesis. The tumor growth factor β (TGF-β) signaling pathway also induces genome rearrangement by reduction of HR DSB repair in human cancer cell lines, leading to increased copy number alterations and resistance to multiple chemotherapeutic drugs [91, 92]. Stress-induced and localized mutagenesis in multicellular organisms and the relevance of these mechanisms to cancer are reviewed in more detail elsewhere [12].

## Evolution and applications of stress-induced mutation

Mutations provide the raw material for evolution but can also decrease the fitness of an organism. Therefore, mutation rates have, presumably, been finely tuned, apparently through second-order selection. Constitutively high mutation rates are advantageous in rapidly changing environments but decrease fitness in more stable (or periodically changing) environments. By biasing mutation to times of stress and to particular genomic regions, perhaps such regions relevant to a specific stress, stress-induced mutagenesis mechanisms provide the benefits of high mutation rate, while mitigating the risks. The ubiquity of these mechanisms throughout the tree of life supports their crucial role in evolution.

Stress-induced mutation mechanisms, first discovered in bacteria, challenge historical assumptions about the constancy and uniformity of mutation but do not violate strict interpretations of the Modern Synthesis. Mutation is still viewed as probabilistic, not deterministic, but we argue that regulated mutagenesis mechanisms greatly increase the probability that the useful mutations will occur at the right time, thus increasing an organism’s ability to evolve and, possibly, in the right places. Assumptions about the constant, gradual, clock-like, and environmentally blind nature of mutation are ready for retirement.

Stress-induced mutation mechanisms are likely to play important roles in human disease by promoting pathogen and tumor evolution and may drive evolution more generally. Mutation mechanisms may also be attractive drug targets for combatting infectious disease, cancer, and drug-resistance evolution in both [73]. Although many mechanisms of stress-inducible mutation have been identified in the past two decades [12], these are likely to be the tip of the iceberg. Some current pressing questions are highlighted below.

## Open questions in mutation research

- What fraction of total “spontaneous” mutagenesis results from mutagenesis up-regulated by stress responses? Do stress response–regulated mutation programs drive much of adaptive evolution in microbes? Multicellular organisms?
- Are DSBs and the mutations they cause randomly distributed in genomic space? Or is DSB formation regulated, biased, or directed? By what mechanisms? Is this targeting adaptive?
- Can stress response–regulated mutation mechanisms be targeted by anti-evolvability drugs that limit the generation of heritable diversity? Can these drugs prevent pathogens and cancers from out-evolving host responses and drugs?
